# Variability in primary productivity determines metapopulation dynamics

**DOI:** 10.1098/rspb.2015.2998

**Published:** 2016-04-13

**Authors:** Néstor Fernández, Jacinto Román, Miguel Delibes

**Affiliations:** Department of Conservation Biology, Estación Biológica de Doñana, Spanish Council for Scientific Research EBD-CSIC, Seville 41092, Spain

**Keywords:** bottom-up population regulation, connectivity, habitat dynamics, ecosystem functioning, Enhanced Vegetation Index, remote sensing

## Abstract

Temporal variability in primary productivity can change habitat quality for consumer species by affecting the energy levels available as food resources. However, it remains unclear how habitat-quality fluctuations may determine the dynamics of spatially structured populations, where the effects of habitat size, quality and isolation have been customarily assessed assuming static habitats. We present the first empirical evaluation on the effects of stochastic fluctuations in primary productivity—a major outcome of ecosystem functions—on the metapopulation dynamics of a primary consumer. A unique 13-year dataset from an herbivore rodent was used to test the hypothesis that inter-annual variations in primary productivity determine spatiotemporal habitat occupancy patterns and colonization and extinction processes. Inter-annual variability in productivity and in the growing season phenology significantly influenced habitat colonization patterns and occupancy dynamics. These effects lead to changes in connectivity to other potentially occupied habitat patches, which then feed back into occupancy dynamics. According to the results, the dynamics of primary productivity accounted for more than 50% of the variation in occupancy probability, depending on patch size and landscape configuration. Evidence connecting primary productivity dynamics and spatiotemporal population processes has broad implications for metapopulation persistence in fluctuating and changing environments.

## 1. Introduction

Ecosystem functioning, understood as the pools and fluxes of matter and energy produced at the ecosystem level, has been claimed to play a key regulatory role in primary consumer populations by determining the net energy flux input into trophic webs [1]. This idea relies on the importance of primary productivity, a major output of ecosystem functioning, in determining the overall quantity and quality of food resources available to herbivores, and ultimately on the role of bottom-up forces in regulating herbivore populations [2]. Furthermore, ecosystem primary productivity has been claimed to be a major link between animal performance and the spatio-temporal variability in the abiotic environment, via trophic interactions [3]. Moreover, herbivore species can develop adaptive physiological and behavioural responses to maximize their fitness according to primary productivity patterns experienced in their habitats; for example, matching their reproduction period to cycles of maximum vegetation activity [4], or adapting their movements to spatio-temporal fluctuations in productivity [5]. Variability in primary productivity may also influence life history and population parameters of animals, such as conception and birth rates, survival and overall population recruitment [6–8]. Thus, primary productivity patterns can play a central role in determining the quality of animal habitats and thereby affect their population dynamics, especially in highly fluctuating environments where animals need to track energy pulses and bottlenecks [9,10].

It remains unclear how temporal fluctuations in primary productivity and, more generally, fluctuations in the habitat quality may determine the dynamics and distributions of spatially structured populations. A distinctive characteristic of these populations is that habitat occupancy rates can be highly dynamic depending on the balance between local extinction and recolonization processes in networks of habitat patches [11]. Factors affecting spatially structured populations have motivated extensive research within the framework of the metapopulation paradigm, with a strong focus on how habitat occupancy patterns and colonization–extinction processes respond to the interacting effects of the local habitat characteristics and the structure of the landscape. Yet the influence of spatial and temporal habitat heterogeneity has received uneven consideration in this context [12]. Early observational studies often made simplifying distinctions only between suitable habitats and the non-habitat, which assumes that the size and the spatial distribution of the habitats contained the most significant information for predicting occupancy rates. However, much empirical evidence on real-world metapopulations has later demonstrated that habitat quality is bound to affect all processes determining metapopulation dynamics, from local dynamics to spatial connectivity [13–18]. Differences in habitat quality can have similar or greater influence on occupancy rates than the size and the distribution of the habitats, implying that accounting for the environmental causes of habitat heterogeneity is critical for understanding metapopulation persistence [19]. Beyond spatial habitat heterogeneity, existing theory and experiments also predict that temporal variability in the quality of the habitat is key to the dynamics of many real metapopulations, and that metapopulations are significantly more vulnerable to stochastic extinction risks in fluctuating, rather than constant, environments [20–22]. However, empirical metapopulation studies have largely overlooked habitat-quality fluctuations.

**Figure 1.**
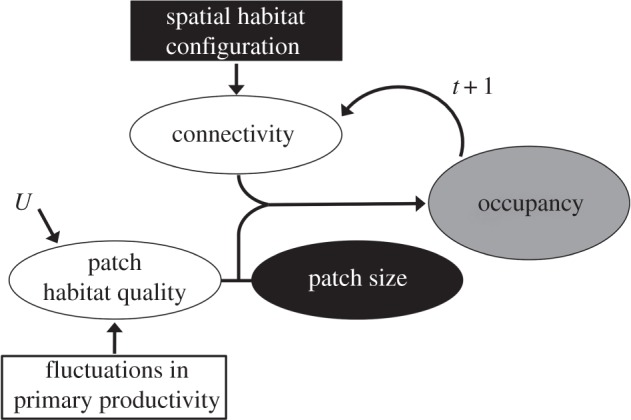
Conceptual diagram showing how herbivore habitat occupancy dynamics may be affected by the interaction between the bottom-up effects of variability in primary productivity and the structure of the habitat network. The classical metapopulation model emerged focusing on the influence of the geometry of the habitat network on metapopulation dynamic processes (black boxes), and assuming that the local population-size effects can be captured by habitat size. However, variability in habitat quality can also cause differences among patches in the occupancy probability, such as through affecting the local probability of extinction and the conditions for the attraction and settlement of immigrants. Through determining the amount of energy available to primary consumers, temporal variations in primary productivity would also produce variability in the habitat quality, affecting occupancy at time *t* and propagating through the occupancy–productivity relationship in time *t* + 1. *U* denotes other unknown sources of extrinsic variability affecting the quality of the habitats.

Temporal environmental variability is expected to increase the complexity of habitat effects on the demography of metapopulations in at least two different ways (figure 1). First, environmental variability can generate fluctuations in the quality of habitat patches (e.g. through altering the levels of trophic resources). It is expected that these fluctuations would cause variability in the local colonization and extinction probabilities. Second, changes in habitat quality imply that the spatial pattern of connections between patches may change over time and potentially produce a temporal decoupling between patch connectivity and occupancy probability. For example, the transient time of recovery after new connections are established may cause lower patch occupancy levels in fluctuating environments than what would be expected in a static landscape [23,24]. The key role of habitat dynamics in driving metapopulation dynamic processes has been shown in successional habitats, in which have been observed an overall reduction in patch occupancy levels and a less clear connectivity–occupancy relationship as compared with ‘static’ landscapes [25].

We used a unique long-term dataset on patch occupancy patterns in a metapopulation of *Arvicola sapidus*, a strictly herbivore rodent, to test the hypothesis that the bottom-up effects from variability in primary productivity regulates the dynamics of spatially structured herbivorous populations. To the best of our knowledge, this is the first time this hypothesis has been tested. Specifically, we predicted that the dynamics of patch occupancy in the metapopulation would be linked to temporal variability in the rates and seasonal course of annual primary production. We analysed the metapopulation–productivity relationship in a highly fluctuating Mediterranean ecosystem, given that we expected that populations would be particularly responsive in highly stochastic environments. Primary productivity patterns in Mediterranean ecosystems are typically characterized by pronounced seasonality and high inter-annual variability [26,27]. Whereas the vegetation growth period mainly occurs between autumn and spring, productivity dramatically decreases as a result of moisture deficits during summer drought periods, which vary considerably in their duration and intensity.

We analysed spectral remote sensing satellite data to characterize inter-annual variability in the course of primary production. These data provide systematic, spatially comprehensive and long-term information on vegetation activity and the energy and matter exchange at the ecosystem level, which ultimately determines the rate of energy input into trophic webs [28–30]. Thus, remote-sensing information is of great value in overcoming some of the practical difficulties that have traditionally challenged the analysis of temporal habitat variability in animals that may result from the changing availability of nutritional resources [9].

We specifically evaluated the following hypotheses and predictions:

(1) Primary productivity regulates spatially structured herbivore populations through classic bottom-up processes, determining the amount of energy available for consumption. At the level of habitat patches, this would manifest as increased habitat quality during years with high vegetation productivity. Thus, we predicted that total annual primary productivity and within-year variability in primary productivity would have a positive and negative effect, respectively, on the species incidence in habitat patches.
(2) Spring productivity determines the amount of energy available prior to a pronounced food shortage period for herbivores in Mediterranean ecosystems. This period also marks the end of the breeding season in many herbivore species, and therefore population growth may be limited by the timing of the end of the productivity season. We expected temporal dynamics in patch occupancy, colonization and extinction to be affected by the total amount of primary productivity in spring, and by variability in the phenology of productivity as defined by the total duration of the growing season and the timing of its end.
(3) Connectivity plays a key role in metapopulations by determining the probability of patch colonization by migrants from other patches. Thus, fluctuations in patch occupancy may not only depend on local habitat quality but also on the quality of surrounding patches and their capacity to produce emigrants. We studied delayed connectivity effects under the hypothesis that species incidence is affected by primary productivity in surrounding patches during the previous year. Specifically, we tested the prediction that changes in connectivity rates, as determined by the previous year's primary productivity, would have an additive effect on patch occupancy dynamics.

## 2. Material and methods

### (a) Study metapopulation

The southern water vole is a medium-sized arvicoline rodent ranging throughout France and the Iberian Peninsula. It is a strictly herbivorous species and its habitat has been traditionally described as permanent water courses and ponds with high availability of riverine herbaceous plants that provide feeding and shelter. However, populations in Mediterranean environments may also be found in temporary wetlands subject to drought periods, some of which may even be prolonged [31].

The study metapopulation was located in the Doñana Protected Area in southwestern Spain (37°00′ N, 6°30′ W). The area is situated at the sea level and has a Mediterranean sub-humid climate with well-defined seasonality, having hot and dry summers, and moderately wet and mild winters. Mean annual rainfall is 550 mm, although there is great variability between years (5th–95th percentiles for the period 1984–2014 = 252–946 mm). Most (79%) of the annual precipitation occurs between October and March. In Doñana, the temporal precipitation distribution is the main influence on the seasonal course of vegetation activity. Aboveground primary production typically shows a sharp increase from autumn to winter followed by a gradual decrease in spring [27].

The habitat of *A. sapidus* in the area is highly patchy and consists of temporary ponds, most of which are smaller than 1 ha, embedded in a matrix of shrubland formations [31]. Vegetation mostly consists of helophytes and perennial herbaceous communities dominated by *Juncus* spp., *Scirpus* spp., *Imperata cylindrica* and *Agrostis stolonifera* and sometimes also with the presence of *Erica scoparia* shrubs and *Rubus ulmifolius* thickets [31]. No genetic evidence of dispersal barriers has been found for the species within the study area [32].

Female southern water voles are polyestrous and can start breeding at the age of four months. Reproduction mainly occurs between November and May and reaches its highest between February and April. Breeding stops during the drought season probably as a result of food shortage. Patch population sizes are usually low, with a maximum-recorded local population size of 32 individuals and a median density of 7.7 individuals ha^−1^ (*n* = 44 habitat patches surveyed during 135 capture–recapture campaigns between 2000 and 2002; J. Román *et al.* 2016, unpublished).

We monitored the southern water vole in six different study plots within the Doñana Protected Area during 13 years between 2001–2008 and 2010–2014. The study plots consisted of circular areas (3 km diameter). A total of 300 ponds were identified *in situ* at the beginning of the study period and all of them were initially considered to be potential habitat patches (median patch size = 0.18 ha; range = 0.11–10.7). Patch boundaries were digitized in the field using a GPS receiver with precision of less than 5 m.

The metapopulation was monitored by intensively surveying each habitat patch between the end of June and the end of July each year (i.e. during the drought season, when the species does not reproduce). We assessed species occurrence based on the presence of fresh faeces and latrines, which are highly conspicuous. Preliminary tests based on 279 visits that resulted in occupied patches showed 100% detectability within the first 15 min of survey in patches of less than 0.2 ha, and within the first 25 min in larger patches. Thus, we established a searching time of 20 and 30 min for patches smaller or larger than 0.2 ha, respectively. We identified all colonizations and extinctions at the patch level for 11 of the 13 yearly surveys by examining species occurrence in consecutive years (i.e. for all sampling years except 2001 and 2010).

### (b) Variability in primary productivity

Time series of the Enhanced Vegetation Index (EVI) were measured at each study plot as an integrative indicator of aboveground net primary productivity (ANPP). The EVI is a measure of vegetation photosynthetic activity calculated from reflectance in the red, near infrared and blue portions of the electromagnetic band. The normalized infrared/red reflectance ratio is an indicator of the fraction of photosynthetically active radiation intercepted by vegetation, which in turn determines ANPP [33–35]. By using the blue band, the EVI corrects for aerosol effects and is less prone to underlying soil influence and signal saturation [36].

Variability in primary productivity was characterized at each site using the following variables: the annual integral of EVI (iEVI), a proxy for total annual ANPP; the within-year variability in EVI (cvEVI), which captures variations in ANPP (e.g. associated with seasonality); the length of the growing season (LOS) and the date of its end (EOS) as indicators of ecosystem phenology; and the spring EVI (spEVI) as a measure of productivity during the period in which *A. sapidus* reproduction is at its highest, but prior to drought. These variables were calculated using a 14-year series of composite (16 days) images acquired by the MODIS Terra sensor at a spatial resolution of approximately 240 m (MOD13Q1 Collection 5). Initially, the images were filtered on a per-pixel basis using the accompanying reliability and quality assessment information. Pixels that could have been contaminated by clouds, shadows and high levels of atmospheric aerosols were eliminated. The aggregated EVI time series for each study plot for each date were derived by calculating the median value of all valid pixels intersecting the plots. Time series were finally smoothed using a cubic smoothing spline with quadratic weight in order to remove spikes while compensating for higher EVI values, which are usually less affected by noise in the data.

The iEVI and cvEVI were calculated as the sum and the coefficient of variation, respectively, of all EVI values for the period between summer and spring immediately before each survey, taking 1 June as the start of summer and 31 May as the end of spring. The start of the growing season (SOS) was calculated as the date of the year when the midpoint between the minimum EVI and the next maximum was reached during the annual phase of productivity increase [37]. The EOS was calculated in the same way for the midpoint between the maximum EVI and the next minimum during the decay phase. LOS was the difference (in days) between SOS and EOS. To avoid redundancy, only LOS and EOS were analysed. Finally, spEVI was calculated as the sum of all the EVI values between March and May.

### (c) Statistical analyses

We evaluated metapopulation responses to ecosystem fluctuations using generalized linear mixed models (GLMM) with binomial distribution and model selection protocols based on the Akaike information criterion (AIC) [38,39]. Three metapopulation parameters measured during summer were modelled under the same scheme: patch occupancy, colonization and extinction.

Two subsets of models were defined *a priori*. The first subset included two models designed to test the prediction that variability in the annual aboveground primary productivity determines patch occupancy dynamics. The simplest model included iEVI as the only primary productivity effect, and the other model included cvEVI to account for the heterogeneous distribution of productivity within the year (table 1). The second subset was designed to evaluate the prediction that variability in phenology and spring productivity determines occupancy dynamics. This subset consisted of three models: the simplest one included the effect of the spring productivity integral (spEVI), and the more complex models included the effects of EOS and LOS. All tested models included also patch area—often used as a surrogate of potential population size in metapopulation studies [11]. In addition, we specified a saturated and a null model. The saturated model included all variables from the two subsets representing the combined effects of integrated annual productivity and phenology variability, excluding variables highly correlated with others (*r* > 0.5). The null model only included the area as a reference to determine the mean expected response under the assumption of no effects of temporal variability in primary productivity. All models included the same random component structure with the patch nested within the study plot to model repeated measurements, and an exponential spatial covariance structure to correct for potential random spatial autocorrelation [40]. Finally, fitted GLMMs were compared based on differences in AIC and model probabilities calculated from the Akaike weights (*w_i_*) [38].

**Table 1.** Selected generalized linear mixed models for the effects of the dynamics of primary production and connectivity during the previous year on water vole occurrence, colonization and extinction for 2002–2008 and 2010–2013.

model	estimate	standardized	*t*	*R*^2^_GLMM(m)_	*R*^2^_GLMM(c)_
occurrence (*n* = 1740)				0.41	0.56
intercept	−6.77 ± 0.96	−0.90	−7.03		
area	0.46 ± 0.08	1.44	5.55		
spEVI	4.76 ± 1.71	0.22	3.14		
EOS	0.23 ± 0.05	0.33	2.78		
*S*_*i*(*t* −1)_	0.19 ± 0.04	0.44	4.17		
colonization (*n* = 1082)				0.14	0.23
intercept	−12.17 ± 1.99	−1.82	−6.12		
area	0.31 ± 0.09	0.96	3.59		
iEVI	21.50 ± 5.99	0.42	3.59		
cvEVI	−2.58 ± 1.61	−0.28	−1.60		
EOS	0.23 ± 0.07	0.32	3.60		
LOS	0.03 ± 0.03	0.14	1.02		
*S*_*i*(*t* −1)_	0.15 ± 0.07	0.26	2.16		
extinction (*n* = 513)				0.47	0.51
intercept	1.29 ± 0.78	−0.43	1.65		
area	−0.33 ± 0.08	−0.99	−4.11		
spEVI	−4.27 ± 2.65	−0.20	−1.61		

In a second step, we analysed the lagged effects of ecosystem productivity on occupancy dynamics that may result from changes in connectivity through time. This analysis attempted to capture the effect of the previous year's habitat quality on patch connectivity and occupancy (i.e. assuming that the more connections to high-quality patches at time *t* − 1, the higher the probability of finding the species at *t*). Thus, we calculated a patch connectivity index each year using a modified version of the negative exponential kernel function [11] in which the contribution of surrounding patches was weighted by the previous year's habitat quality:2.1
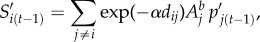
where *d_ij_* is the shortest distance between the outer boundaries of the focal patch *i* and each surrounding patch *j*; *α* is a scale parameter defined by the mean dispersal distance from the population dispersal kernel (here denoting the distribution of distances between post-dispersal locations and the source location [41]); *A_j_* is the area of patch *j*; *b* defines the power relationship between patch area and species abundance; and 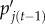 is the probability of patch occupancy at year *t* − 1. This probability was calculated using the predictions of the best occupancy model obtained from previous analyses. Therefore, 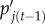 reflects the expected effect of primary productivity conditions of the previous year through modifying the habitat quality of surrounding patches. We set *α* = 1/665 based on a previous isolation-by-distance genetic study of dispersal distances, which resulted in mean dispersal distances of 668 m for males and 661 m for females. These estimates were obtained using the average squared axial parent–offspring distance estimated on the regression of individual pairwise genetic distances on the geographical distances [32]. In addition, we calculated *b* = 0.27 based on a power-law relationship between patch size and the mean number of individuals calculated in 44 habitat patches surveyed during 135 capture–recapture campaigns held between 2000 and 2002 (*n* = 2662 captures of 928 different individuals in 7703 trap-nights with a recapture rate greater than 0.98; after [42]). Finally, 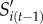 was estimated by including also all patches within a 665 m radius from each patch coinciding with the 50% accumulated dispersal probability from the dispersal kernel. We restricted the buffer to this radius in order to be able to compare the results with connectivity estimates based on recorded occupancy data in the patch neighbourhood (see below). Ponds outside the study plots were identified using two fine-scale digital maps of the flooding areas in the region [43,44].

We assessed the effect of the connectivity index on patch occupancy dynamics by comparing models with and without 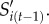 Furthermore, in order to test for the specific effect of primary productivity fluctuations on connectivity we also tested competing models where this ‘dynamic-habitat’ connectivity index 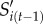 was replaced by a ‘static-habitat’ index 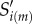 in which 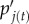 was set constant. The latter represents a situation where the contribution of sources only depended on their size and spatial distribution and not on variations in primary productivity. For these analyses, we only considered models previously supported with probabilities *w_i_* ≥ 0.05. The year 2001 was excluded from these analyses as 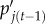 could not be estimated due to incomplete MODIS data for the previous year.

Last, we tested the predictive capacity of the dynamic-habitat and the static-habitat connectivity indices against the original incidence–function connectivity *S_i_*; that is, including *p_j_* (=0 for unoccupied and 1 for occupied) instead of 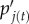. These relationships were tested using linear mixed models with the patch as a random term and specifying an exponential variance structure to account for higher variance at larger connectivity values. This test was performed for a subset of 38 habitat patches and 11 years where the patch occupancy status by *A. sapidus* was known for all surrounding patches within a 665 m radius.

Statistical analyses were performed with the R statistical environment v. 3.0.2 [45], except for GLMM with spatial random structures, which were fitted in the SAS statistical package [46]. Pseudo-*R*^2^ statistics were estimated as an approximate measure of the model's goodness of fit, separating the marginal component (i.e. variability explained by the fixed factors; 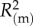) and the conditional component (i.e. variability explained by both fixed and random factors; 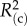) [47].

## 3. Results

### (a) Patch occupancy patterns

In total, 145 of the 300 potential habitat patches were occupied by *A. sapidus* at least once during the 13-year study. Subsequent analyses focused only on patches in which presence was recorded at least once. On average, 32.6% of the 145 patches were occupied each year (range 16–56%). There were 614 recorded occurrences and 1271 recorded absences in patches that had been occupied at least once. We observed 166 colonizations of 1082 possible events, and 186 extinctions of 513. Only three patches were permanently occupied.

### (b) Variability in primary productivity

The analysis of the EVI time series confirmed the great variability in annual and seasonal rates of aboveground primary production, as well as in phenology variables; differences in productivity within study sites ranged between 18.3% and 41.1% for iEVI, and between 17.7 and 71.3% for spEVI. Within-site variations in LOS varied between 128 and 288 days, and the difference in EOS ranged between 48 and 96 days. There was a high correlation between iEVI and spEVI (Pearson's *r* = 0.68), cvEVI and spEVI (*r* = 0.83), and cvEVI and esEVI (*r* = 0.77). All other pairs had *r* < 0.5. The combined effects model thus excluded cvEVI and spEVI.

### (c) Patch occupancy, colonization and extinction models

Results of model selection supported the hypothesis that inter-annual variability in seasonal primary productivity determined metapopulation dynamics. We found very high support for a model relating the patch occupancy probability by *A. sapidus* with higher spring productivity (spEVI) and a later end of the growing season (EOS) (*w_i_* = 0.99; electronic supplementary material, table S1-A). The colonization model with the highest probability was the combined-effects model (*w_i_* = 0.95) showing a positive effect of long and late-ending growing seasons (LOS and EOS), as well as positive and negative effects of iEVI and cvEVI, respectively. Last, extinction analyses supported the seasonal hypothesis but were less conclusive. The highest-ranked model included spEVI (*w_i_* = 0.54), although the probability of the null ‘patch-area’ model was also high (*w_i_* = 0.46; electronic supplementary material, table S1-A).

### (d) Connectivity effects

**Figure 2.**
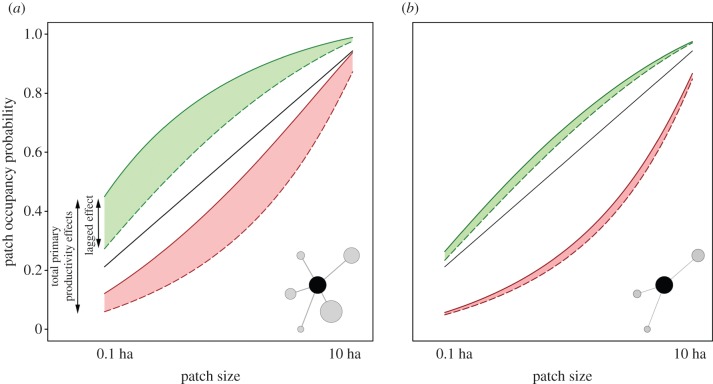
The predicted effects of temporal variability in primary productivity on patch occupancy dynamics. (*a*, *b*) The patch occupancy probability for two different landscapes differing in the maximum connectivity potential. Predictions are shown for four different primary productivity scenarios in relation to the focal patch area. The green region shows the predictions for a ‘good’ year (i.e. with high spring productivity and a later end of the growing season), with a prediction region defined by the variability in previous year conditions. The upper (solid) bound and the lower (dashed) bound correspond to the predicted probability for a ‘good’ year preceeded by another ‘good’ year or by a ‘bad’ year, respectively. Similarly, the red area represents the patch occupancy probability for a ‘bad’ year (i.e. with low spring productivity and an earlier end of the growing season), and is also bounded by predictions when the preceeding year is’ good’ (solid) or ‘bad’ (dashed). The solid line corresponds to the predictions from the null model incuding only patch size as a predictor without considering primary productivity effects. Prediction curves correspond to fixed parameter values at the 90th and 10th percentiles of all observed values for the total spring productivity (spEVI), date of end of the growing season (EOS) and maximumm potential connectivity (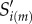).

Static- and dynamic-habitat connectivity were tested through adding 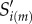 and 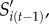 respectively, in models supported with probabilities ≥0.05 in the previous analyses (electronic supplementary material, table S1-B). The additive effect of 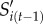 on the metapopulation dynamics was clearly supported by occupancy analyses (*w_i_* = 0.97). The predictions from the final selected model (table 1) indicate that the cumulative effects of variability in seasonal productivity in two successive years have a strong impact on patch occupancy probability (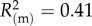), especially in landscapes with a high number of potential connections to other patches (figure 2).

The separate analysis of colonization and extinction supported the effect of the dynamic connectivity on colonization probability (*w_i_* = 0.57; table 1; electronic supplementary material, table S1-B). The inclusion of 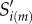 instead of 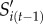 always resulted in poorer models, thus confirming the primary role of the course of primary productivity at *t* − 1. On the other side, effects on extinction were highly uncertain; although the highest-ranked extinction model included 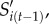 all the competing models showed a similar degree of support (electronic supplementary material, table S1-B).

**Figure 3.**
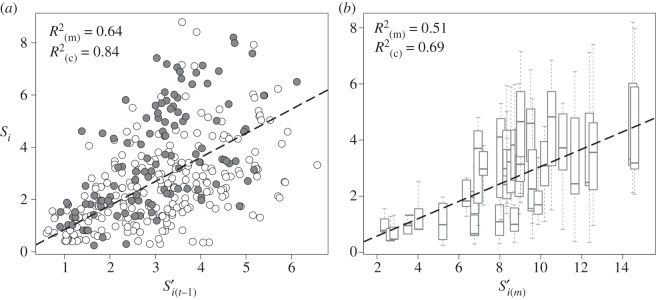
The relationships between the two connectivity metrics evaluated in this study and the incidence function connectivity index (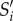) [11]. Metrics were compared using data from 38 patches and 11 years and accounting for patches within a 665 m radius. Dots in (*a*) represent 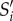 versus the dynamic connectivity (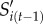) metric for each patch and year. Closed and open dots represent occupied and unoccupied patches, respectively. Boxplots in (*b*) show the distribution of *S_i_* measured at each patch against the static connectivity metric 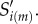

Figure 3 shows the results of fitting *S_i_* against 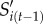 and 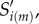 respectively, for the subsample of 38 habitat patches. Linear mixed models confirmed that both relationships were significant (*p* < 0.001) although weighting landscape connectivity by productivity fluctuations captured better the spatial pattern of connections among occupied patches (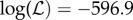) than the ‘static-habitat’ connectivity index (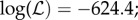 ΔAICc = 55.0).

## 4. Discussion

We found support for the hypothesis that spatio-temporal population dynamics were linked to inter-annual variability in the course of primary productivity, and specifically in the seasonal levels and duration of the productivity pulse preceding the summer energy bottleneck period. This finding was best captured by the habitat occupancy analyses (table 1): productivity in spring and the date of the end of the growing season explained temporal variations in the incidence of *A. sapidus* within the habitat network.

The results are important because they show that fluctuations in ecosystem functioning can play a major role in regulating the habitat quality of spatially structured populations. Food limitation has been postulated as a key control on the rates and temporal distribution of reproduction and offspring survival in small herbivores, especially in rodents [48–50]. In addition, the course of primary productivity in Mediterranean ecosystems typically displays pronounced seasonality associated with the decoupled distribution of precipitation and high temperatures, the summer water deficit being the critical control of vegetation activity. Thus, a later end of the productivity season extends the favourable habitat conditions for the reproduction of herbivores before the summer bottleneck. This extension would have particularly important effects on the population growth of fast-living species with early sexual maturation like *A. sapidus* [42], resulting in a higher number of newborn individuals reaching sexual maturation within the same breeding season, and thus in a larger reproductive pool. Therefore, our finding that habitat occupancy increases with a later-ending and more productive growing season could be explained by at least two processes. First, an increased breeding population would decrease the local probability of stochastic extinctions in occupied patches. Second, an overall enhancement of habitat conditions would favour the colonization of empty habitats, a process that depends on both the production of potentially dispersing individuals in the habitat network and the local habitat conditions being of sufficient quality to allow them to become established. Colonization analyses supported this second prediction, showing that patch colonization probabilities were determined by the length of the time window of favourable conditions that allow for reproduction, offspring dispersal and settlement before the productivity bottleneck. Although extinction analyses pointed out to lower extinction probabilities in years with higher productivity in spring, they were less conclusive and we could not rule out an alternative model linking local extinctions exclusively to patch size. Last, our results also reveal the importance of monitoring ecosystem phenology from remote sensing for tracking habitat quality fluctuations in relation to trophic limitation, an idea that is also supported by increasing evidence connecting directly spectral vegetation indices with the diet quality and composition of herbivores [51].

This study provides empirical evidence on the effects of habitat dynamics on connectivity in a natural metapopulation. We found that accounting for the course of primary productivity in the previous year significantly increased the capacity of connectivity metrics to predict habitat colonizations and occupancies. Furthermore, the dynamic connectivity index (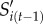) fitted better the incidence–function connectivity index—as calculated from observed occupancies (*S_i_*)—than an alternative static-landscape index calculated only on the basis of the size and spatial arrangement of the habitats (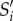). In other words, the effects of connectivity were best captured by the interactions between habitat-quality fluctuations and the geometry of the network (figure 1). It must be emphasized that the connectivity–occupancy relationship has been scarcely examined for natural metapopulations in fluctuating environments, despite this relationship is the defining driver of the dynamics of metapopulations. However, using simulation modelling, Ellner & Fussman [20] showed that failing to incorporate a ‘patch-dynamics’ perspective had major consequences for predicting metapopulation persistence in the context of successional habitat dynamics. Similarly, Visconti & Elkin [52] advocated for the inclusion of habitat-quality estimates into connectivity metrics in order to determine the contribution of individual patches to metapopulation persistence in fluctuating environments. Hodgson *et al*. [25] cautioned that metapopulation studies based on time-averaged or snapshot species distribution data may actually underestimate the effect of connectivity on occupancy patterns, because they overlook potentially important changes in the connectivity structure of the network. Our results not only confirm the importance of accounting for temporal habitat heterogeneity for predicting connectivity; they also illustrate a probably common phenomenon in nature in which temporal changes in connectivity may emerge from typical environmental variability affecting the quality of the patches.

The relationship between previous-year primary productivity and occupancy probability also points out a mechanism for carry-over effects on the population dynamics: the rate of patch occupancy was influenced by habitat dynamics operating for at least two consecutive years. This effect entails a dramatic increase in the predicted occupancy rates in a given habitat in time (figure 2). For some landscape configurations, we predicted within-patch differences in the probability of habitat occupancy of up to Δ*p* > 0.5 depending on whether the two previous years were ‘good’ or ‘bad’, respectively, in terms of primary productivity (i.e. area between upper green and lower red lines in figure 2). Hence, cycles of favourable or unfavourable conditions maintained over 2 years would dramatically amplify local population growths and declines. This has important consequences for predicting metapopulation persistence. For example, climate projections indicate that rising temperatures will result in shifts in vegetation phenology in Mediterranean environments, with earlier and longer-lasting summer drought periods [53,54], meaning that populations of primary consumers like *A. sapidus* would be subject to longer periods of lower habitat-quality conditions, making them more vulnerable to extinction. We argue that a better understanding on the relationships between climate patterns, variability in primary productivity and consumer population regulation is paramount for predicting the propagation of climate change effects through trophic regulation mechanisms.

In this study, we have focused on the bottom-up effects of variability in primary productivity on a small herbivore metapopulation. It is well known that bottom-up processes affecting small mammals can be severely modified by top-down regulation from predators [55]. Indeed, experimental studies in different vole species have shown that introduced predators can suppress population growth in local habitats through interfering with trophic regulation processes. However, the impact of this local suppression on the colonization–extinction dynamics at the metapopulation level were less obvious [56,57]. We found that local extinction events were strongly associated with habitat size, whereas the effect of primary productivity fluctuations was comparatively much smaller. It can be speculated that, with more than 30 potential predator species sporadically consuming *A. sapidus* [58], predation could have a significant impact on the local probability of extinction depending on the habitat size, as the extirpation of fewer individuals would drive extinction in smaller populations. However, assessing simultaneously the temporal effects of top-down and bottom-up forces on spatially structured populations remains highly challenging, especially in natural systems with different species of coexisting predators.

## 5. Conclusion

We proposed a novel approach for assessing habitat dynamics and bottom-up regulation processes in the context of spatially structured populations by examining the variability in ecosystem-level indicators related to the rates and phenology of primary productivity. Using long-term metapopulation data, we showed that fluctuations in primary productivity can dramatically increase the variability of habitat occupancy rates in fragmented landscapes, an aspect that a more traditional focus on networks of (presumably) static habitat patches would have not detected. The course of primary productivity preceding the energy bottleneck period also determined annual variations in the degree of connectivity to other suitable patches, suggesting the existence of lagged metapopulation responses to previous year's conditions. Understanding the effects of primary productivity fluctuations on fragmented herbivore populations is important because they link potential changes in species habitats with ecosystem degradation processes, such as those resulting from climate change.

## Supplementary Material

Supplementary Table S1

## Data accessibility

The datasets supporting this article can be accessed in the following digital repository: http://dx.doi.org/10.5061/dryad.vn66m.
