# Supplementary material for: Variability in primary productivity determines metapopulation dynamics
Source: Proc Biol Sci. 2016 Apr 13;283(1828):20152998. doi: 10.1098/rspb.2015.2998 (PMC4843648; doi:10.1098/rspb.2015.2998)
Supplement: Supplementary Table S1 [file rspb20152998supp1.pdf]

VARIABILITY IN PRIMARY PRODUCTIVITY DETERMINES METAPOPULATION DYNAMICS

Néstor Fernández, Jacinto Román & Miguel Delibes

Corresponding author. E-mail: nestor@ebd.csic.es

SUPPLEMENTARY MATERIAL

TABLE S1. Model selection results of the Generalized Linear Mixed Models for patch occupancy, colonization, and extinction dynamics in the metapopulation of *A. sapidus* in relation to primary productivity variables. Random components included the habitat patch identity and an exponential spatial covariance structure nested within the study plot. Values in bold correspond to the best-supported model in each case. log($\mathcal{L}$) is the log-likelihood estimate for the model; ΔAICc is the difference between the model AICc and the AICc of the best model; and w_i is the model probability calculated as the Akaike weight. Model probabilities were calculated excluding models differing from their nested counterparts in the inclusion of one single "pretending" variable, i.e. where the more complex model does not improve model fit [45].

A. Primary productivity models		Occupancy (n =1885)			Colonization (n = 1082)			Extinction (n = 513)		
	K	log($\mathcal{L}$)	ΔAICc	w_i	log($\mathcal{L}$)	ΔAICc	w_i	log($\mathcal{L}$)	ΔAICc	w_i
Null										
Area	4	-1059.3	38.6	<0.001	-455.6	28.3	<0.001	-316.0	0.6	0.426
Variability in annual productivity										
Area + iEVI	5	-1059.2	40.5	<0.001	-451.3	21.6	<0.001	-315.9	2.34	–
Area + iEVI + cvEVI	6	-1059.2	42.5	–	-444.1	9.3	0.010	-315.4	3.4	–
Variability in seasonality										
Area + spEVI	5	-1053.9	29.8	<0.001	-452.1	23.2	<0.001	**-314.8**	**0**	**0.574**
Area + spEVI + EOS	6	**-1038.0**	**0**	**0.986**	-443.7	8.3	0.015	-314.4	1.4	–
Area + spEVI + EOS + LOS	7	-1038.0	2.0	–	-442.1	7.2	0.026	-313.9	2.6	–
Combined effects										
Area + iEVI + cvEVI + EOS + LOS	8	-1040.2	8.5	0.014	**-437.4**	**0**	**0.95**	-315.6	7.9	–

B. Primary productivity and connectivity		Occupancy (n =1740)			Colonization (n = 1082)			Extinction (n = 513)		
	K	log($\mathcal{L}$)	ΔAICc	w_i	log($\mathcal{L}$)	ΔAICc	w_i	log($\mathcal{L}$)	ΔAICc	w_i
Null										
Area	4							-316.1	0.8	0.139
Area + $S'_{i(m)}$	5							-314.7	0	0.179
Area + $S'_{i(t-1)}$	5							-314.9	0.5	0.146
Variability in seasonality										
Area + *spEVI*	5							-314.8	0.24	0.188
Area + *spEVI* + $S'_{i(m)}$	6							313.8	0.33	0.170
Area + *spEVI* + $S'_{i(t-1)}$	6							-313.6	0.19	0.179
Area + *spEVI* + *EOS*	6	-923.5	8.8	0.012						
Area + *spEVI* + *EOS* + $S'_{i(m)}$	7	-922.0	7.8	0.019						
Area + *spEVI* + *EOS* + $S'_{i(t-1)}$	7	**-918.1**	**0**	**0.969**						
Combined effects										
Area + *iEVI* + *cvEVI* + *EOS* + *LOS*	8				-437.5	2.58	0.156			
Area + *iEVI* + *cvEVI* + *EOS* + *LOS* + $S'_{i(m)}$	9				-435.9	1.52	0.278			
Area + *iEVI* + *cvEVI* + *EOS* + *LOS* + $S'_{i(t-1)}$	9				**-435.3**	**0**	**0.566**			
